# The *IFNG* rs1861494 Single Nucleotide Polymorphism Is Associated with Protection against Tuberculosis Disease in Argentina

**DOI:** 10.3390/genes9010046

**Published:** 2018-01-22

**Authors:** Agustín Rolandelli, Joaquín M. Pellegrini, Nicolás O. Amiano, María C. Santilli, María P. Morelli, Florencia A. Castello, Nancy L. Tateosian, Alberto Levi, Nicolás Casco, Domingo J. Palmero, Verónica E. García

**Affiliations:** 1 Facultad de Ciencias Exactas y Naturales, Departamento de Química Biológica, Pabellón II, Universidad de Buenos Aires, 4°piso, Intendente Güiraldes 2160, Ciudad Universitaria (C1428EGA), Buenos Aires, Argentina; rolandelliagus@gmail.com (A.R.); joaquinmpellegrini@gmail.com (J.M.P.); nicolasamiano@hotmail.com (N.O.A.); mariaceciliasantilli@gmail.com (M.C.S.); maria.paula.morelli@gmail.com (M.P.M.); florencia.castelloz@gmail.com (F.A.C.); nantateosian@gmail.com (N.L.T.); 2 Instituto de Química Biológica de la Facultad de Ciencias Exactas y Naturales (IQUIBICEN), CONICET-Universidad de Buenos Aires, 4°piso, Intendente Güiraldes 2160, Ciudad Universitaria (C1428EGA), Buenos Aires, Argentina; 3 División Tisioneumonología Hospital F.J. Muñiz, Uspallata 2272, (C1282AEN), Buenos Aires, Argentina; albertojlevi@gmail.com (A.L.); casconicolas@hotmail.com (N.C.); djpalmero@intramed.net (D.J.P.)

**Keywords:** Interferon gamma, mycobacterium tuberculosis, single nucleotide polymorphism, rs1861494, tuberculosis

## Abstract

Interferon gamma (IFNG) plays a key role during *Mycobacterium tuberculosis* (*Mtb*) infection, and several polymorphisms located in its gene are associated with risk of tuberculosis in diverse populations. Nevertheless, the genetic resistance/susceptibility to tuberculosis in Argentina is unknown. The *IFNG* rs1861494 polymorphism (G→A) was reported to alter the binding of transcription factors to this region, influencing IFNG production. Using a case-control study, we found an association between the AA and AG genotypes and tuberculosis resistance (AA vs. GG: odds ratio (OR) = 0.235, *p*-value = 0.012; AG vs. GG: OR = 0.303, *p*-value = 0.044; AA vs. AG: OR = 0.776, *p*-value = 0.427; AA + AG vs. GG: OR = 0.270, *p*-value = 0.022). Moreover, *Mtb*-antigen stimulated peripheral blood mononuclear cells (PBMCs) from healthy donors and AA carriers secreted the highest amounts of IFNG in culture supernatants (*p*-value = 0.034) and presented the greatest percentage of CD4^+^IFNG^+^ lymphocytes (*p*-value = 0.035), in comparison with GG carriers. No association between the polymorphism and clinical parameters of tuberculosis severity was detected. However, our findings indicate that the rs1861494 single nucleotide polymorphism (SNP) could be considered as a biomarker of tuberculosis resistance in the Argentinean population.

## 1. Introduction

*Mycobacterium tuberculosis* (*Mtb*) infects 2 billon people around the world, causing 10.3 million new active tuberculosis cases and 1.8 million deaths annually [1]. In Argentina, last reports estimated 10,733 cases of tuberculosis, clearly demonstrating the importance of this disease [2]. However, most individuals exposed to *Mtb* do not develop active tuberculosis, suggesting that both host genetic factors and environmental causes might influence the susceptibility to the disease [3].

Efficient activation of cellular immunity is important to establish a protective immune response against intracellular pathogens like *Mtb*, where cytokines play an essential role in the process [4,5]. The immune response elicited after *Mtb* infection is critically dependent on CD4^+^ T cells. In particular, T helper (Th) 1 cells play a crucial function in granuloma formation and clearance of *Mtb* [6,7].

Interferon gamma (IFNG) is a key type 1 cytokine produced primarily by natural killer cells and Th1 lymphocytes, is a crucial mediator of macrophages activation and controlling *Mtb* infection. Especially, IFNG has been shown to activate downstream antimicrobial effector pathways, including inducible nitric oxide synthase (iNOS), IFNG-inducible GTPases, phagosomal maturation and acidification, autophagy and vitamin D receptor signaling [8,9,10,11]. The degree of reduction in IFNG production by peripheral blood mononuclear cells (PBMC) is a marker of disease severity in patients with tuberculosis (TB). Besides, IFNG secretion is lower in patients with the most severe manifestation of tuberculosis [12,13]. Moreover, detection of IFNG produced by T cells is the most widely used method for monitoring immune responses following infection or vaccination [14].

It is expected that some genetic variants of main cytokines that operate during host-pathogen interaction would be associated with a higher resistance or susceptibility to *Mtb* infection. Actually, family-based association studies in leprosy and tuberculosis evidenced the genetic influence in the susceptibility to infectious diseases [15]. Indeed, inherited defects of the Interleukin 12 (IL12)/IFNG pathway are related to Mendelian susceptibility to Mycobacterial disease, a disorder characterized by disseminated mycobacterial infections, denoting the importance of the IL12/IFNG pathway, as well as the relevance of the host genetic background [16].

Single nucleotide polymorphisms (SNPs) are believed to be the main source of variability among humans, especially when they influence gene expression or function depending on their location in the DNA sequence. Moreover, since SNPs are relatively easy to be detected, they are considered as one of the best biological markers in association or case-control studies [15]. It has been described previously that polymorphisms in genes related to cytokine expression could affect the susceptibility to different diseases [17,18,19,20,21,22]. Cytokines play a crucial role in the immune response against several infectious diseases. Therefore, cytokine genes are important targets to be studied. For example, there are many SNPs in cytokine genes reported as possible causes of resistance/susceptibility to tuberculosis. Zhang et al. showed that a functional SNP in the promoter gene encoding Interleukin 6 (IL6) is associated with susceptibility to tuberculosis [23]. Furthermore, among others, SNPs in Interleukin 1β (*IL1B*), *IL12*, *IFNG*, Interleukin 10 (*IL10*) and Tumor Necrosis Factor α (*TNFα*) genes were described in relation with risk to tuberculosis disease [24,25,26]. Recently we have demonstrated that the Interleukin 17A (*IL17A*) rs2275913 SNP is associated with protection to tuberculosis but related to higher disease severity in Argentina [27].

The *IFNG* rs1861494 SNP (G→A) is located within a conserved regulatory region of the third intron of the *IFNG* gene (position +2019). It was evidenced that this SNP introduces a new potential CpG methylation site, resulting in altered transcription factors binding to this region, which might have a functional consequence on *IFNG* expression. Actually, it was reported the association of the A allele with higher levels of IFNG, both in plasma and in stimulated T lymphocytes [28,29]. Until now, there are no studies in the Argentinean population analyzing the association between an *IFNG* SNP and tuberculosis.

Thus, it is important to investigate the role of potential genetic variations in molecules of the immune system that participate in the development of the disease in Argentina. In this work, we investigated the potential association of the *IFNG* rs1861494 SNP and active disease. We also evaluated the functional relevance of this SNP during the immune response of the host against *Mtb* and analyzed its impact on clinical parameters of the disease severity.

## 2. Materials and Methods 

Human immunodeficiency virus (HIV)-uninfected patients with active tuberculosis were diagnosed at the Muñiz Hospital (Buenos Aires, Argentina), based on clinical and radiological data, together with the identification of acid-fast bacilli in sputum and isolation of *Mtb* in culture. Patients recruited were individuals vaccinated with Bacille Calmette Guerin *M. bovis* (BCG) that had no underlying diseases (cancer, diabetes, chronic obstructive pulmonary disease, immune-related diseases and others) at the time the sample was obtained and had received less than 1 week of anti-tuberculosis therapy. Healthy donors (HD) recruited were individuals vaccinated with BCG who lack history of tuberculosis, and with no underlying diseases (cancer, diabetes, immune-related diseases and others) or any pharmacological treatment at the time of recruitment. All HD were tested using the QuantiFERON-TB^©^ Gold In-Tube test (QFT-Qiagen, Hilden, Germany), and only QFT negative individuals were included in this group. Subjects with latent tuberculosis (positive QFT) were excluded from the study. Remarkably, more than 85% of individuals from the HD population were subjects exposed to *Mtb* but not infected with the pathogen (negative QFT). These individuals were recruited from families who had at least one patient living in the same household, having high probability of repeated exposure, making this population an appropriate control for the case-control study [15]. All participants provided a written, informed consent for the collection of samples and subsequent analysis. All methods were carried out in accordance with in accordance with the Declaration of Helsinki. The protocols conducted were approved by the Ethical Committee of the Muñiz Hospital (protocol code 295, year of approval 2011; and protocol code 296, year of approval 2013). All the individuals participating in this study were over 18 years old.

Genomic DNA (gDNA) was extracted from whole blood samples using the Quick-gDNA^TM^ Blood MiniPrep (Zymo Research, Irvine, CA, USA) according to the manufacturer’s instructions. Amplification refractory mutation system-polymerase chain reaction (ARMS-PCR) was used for the rs1861494 SNP genotyping. The ARMS-PCR is based on allele specific amplification of desired fragment using primers corresponding to each allelic variant [30]. The sequences of the primers used were: Allele A specific reverse 5′-AAGTAGGTGAGGAAGAAGCA-3′; Allele G specific reverse 5′-AAGTAGGTGAGGAAGAAGCA-3′; Common forward 5′-CCTTGGTGGCTGAGTTGG-3′. As an internal control, human growth hormone (*HGH*) gene primers (Forward 5′-GCCTTCCCAACCATTCCCTTA-3′, Reverse 5′-TCACGGATTTCTGTTGTGTTTC-3′) were included in every PCR mix to verify successful amplification (440 bp amplicon). The amplification was performed in a Multigene Gradient thermal cycler (LabNet International, Edison, NJ, USA). The conditions included initial denaturation (94 °C for 5 min) following a 35 times cycles of denaturation at 94 °C for 30 s, annealing at 62 °C for 50 s and extension at 72 °C for 45 s each cycle; and final extension at 72 °C for 5 min. The rs1861494 genotypes were assessed from the presence/absence of PCR amplicon (168 bp), corresponding to the specific allele (A/G) on 1.5% agarose gel stained with SYBR Green (Thermo Fisher Scientific, Waltham, MA, USA). All genotypes of the rs1861494 SNP were confirmed by direct sequencing of the amplified *IFNG* gene fragment by Sanger method, and a 100% concordance was obtained among the results obtained from ARMS-PCR and DNA sequencing (Supplementary Figure S1).

Plasma samples were collected by blood centrifugation. In vitro stimulation of cells throughout the study was performed with a cell lysate from the virulent *Mtb* H37Rv strain (*Mtb*-Ag, *M. tuberculosis*, Strain H37Rv, whole cell lysate, NR-14822) of the Biodefense and Emerging Infections Research Resources Repository, National Institute of Allergy and Infectious Diseases, National Institutes of Health (Manassas, VA, USA). Peripheral blood mononuclear cells were isolated by centrifugation over Ficoll-Hypaque and cultured (1 × 10^6^ cells/mL) ± *Mtb*-Ag (10 µg/mL) with RPMI 1640 medium (Gibco, Thermo Fisher) supplemented with 1% l-glutamine, 1% penicillin/ streptomycin, and 10% human serum (Sigma-Aldrich, Saint Louis, MO, USA) for five days. Plasma and supernatant samples were stored at −80 °C until IFNG determination by ELISA (BioLegend, San Diego, CA, USA), following manufacturer’s instructions. For intracellular cytokine staining, monensin (1 µL/mL; Sigma-Aldrich) was added for the last 5 h of culture, and then cells were stained with specific fluorophore-conjugated antibodies against CD4 (BioLegend), and IFNG (eBioscience, Thermo Fisher), prior cell permeabilization with 0.5% saponin.

The genotype and allele frequencies were obtained by direct counting. Hardy–Weinberg (HW) equilibrium was tested between cases and controls separately (Χ^2^ test). Comparisons of the distributions of the allele and genotype frequencies between case and control were performed using the *Χ*^2^ test with Yates correction or Fisher exact test. The association level between the rs1861494 genotypes and the case/control condition was estimated as an odds ratio (OR), with a 95% confidence interval (95% CI), calculated by logistic regression after adjusting for confounding variables (age/ethnicity/sex). An a priori statistical analysis to determine the final sample size was performed with an initial population of 50 HD and 50 TB. The sample size estimation to get a test power of 0.8, using the initial population HD minor allele frequency (MAF) of 0.29 and the TB MAF of 0.44, was of at least 130 individuals in each population. The quantitative data was expressed as mean ± standard error of the mean (SEM), and the Mann–Whitney U test or Analysis of variance (ANOVA) and the Kruskal-Wallis post-test for unpaired and non-parametric samples were used to analyze differences between groups. For categorical variables, the *Χ*^2^ test for homogeneity was performed to compare proportions of subjects between groups. All statistical analysis were performed using GraphPad Prism v7.0 or the R software [31]. *p* values of <0.05 were considered statistically significant.

## 3. Results

### 3.1. Demographic Characteristics of the Populations Studied

In order to investigate the association between the *IFNG* rs1861494 SNP and tuberculosis in Argentina, 175 HD and 201 TB were recruited between 2014 and 2017. Demographic characteristics of both populations are shown in Table 1. The populations under study are comparable in terms of ethnicity and age but displayed different sex proportions.

Considering that these disparities could cause substantial problems for genetic association studies, genotypic frequencies distribution of the *IFNG* rs1861494 SNP in HD and TB populations stratified by ethnicity and sex were calculated (Table 2). Since no differences were found, we concluded that dissimilarities between the percentages of individuals from each sex or ethnicity would not affect the genotype distribution analyzed in each population.

### 3.2. The IFNG rs1861494 SNP as a Biomarker for Tuberculosis Protection in Argentina

**Figure 1:**
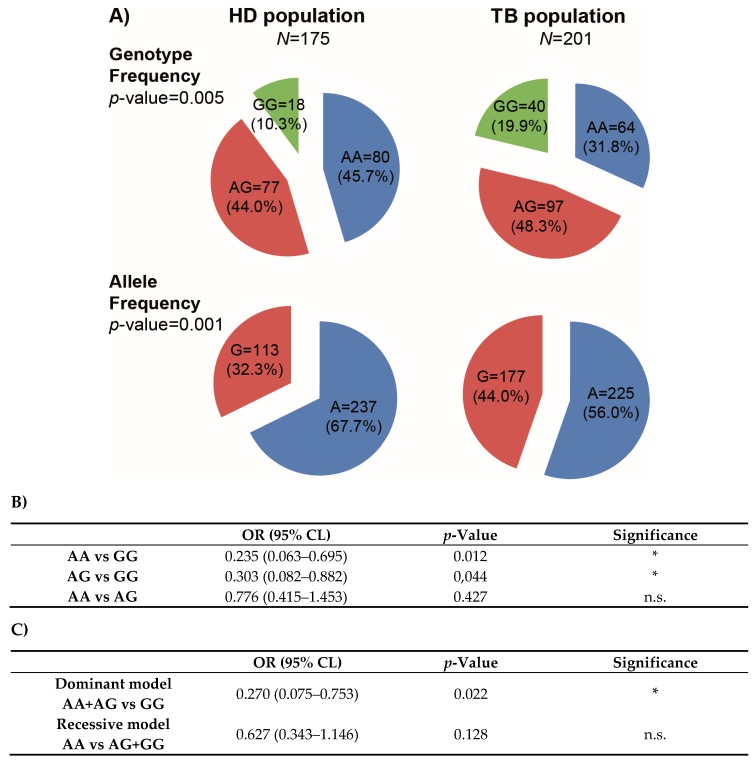
Genotypic and allelic frequencies of the Interferon gamma (*IFNG*) rs1861494 single nucleotide polymorphism (SNP) in healthy donors (HD) and tuberculosis patients (TB) populations in Argentina. (**A**) Pie chart representing the genotypic and allelic distribution of the *IFNG* rs1861494 SNP in both populations. The number of individuals of each population and the frequencies (in parentheses) are detailed. *p*-values were calculated by the Χ^2^ test of homogeneity. Both populations were in Hardy-Weinberg (HW) equilibrium. (**B**) Odds ratios (OR) calculation was used to quantify the association between tuberculosis and the different genotypes. (**C**) Dominant and recessive genetic models for the association between the ra1861494 SNP and TB. Odds Ratio were calculated by logistic regression after adjusting for confounding variables (age/ethnicity/sex).

Figure 1A shows the genotypic and allelic frequencies distributions that were found in HD and TB populations. Importantly, both populations were in HW equilibrium. *Χ*^2^ test of homogeneity showed that HD and TB populations were significantly different regarding the genotypic and allelic frequencies. Both the A allele and the AA genotype were found in a lower proportion in TB population, suggesting that these variants could be associated with tuberculosis protection (67.7% A allele frequency and 45.7% AA genotype frequency in HD population; vs. 56.0% A allele frequency and 31.8% AA genotype frequency in TB population). In fact, OR were calculated to estimate the level of association between the *IFNG* rs1861494 genotypes and tuberculosis disease by logistic regression after adjusting for confounding variables (Figure 1B). By comparing the AA genotype against GG, the OR value was of 0.235 (95% CI = 0.063–0.695; *p*-value = 0.012), indicating an association of the AA genotype with tuberculosis resistance. Additionally, the OR value of 0.303 (95% CI = 0.082–0.882; *p*-value = 0.044) obtained by comparing the AG genotype against the GG, indicated that this genotype could also be associated with tuberculosis resistance. Besides, by comparing the AA genotype with the AG, the OR value was of 0.776 (95% CI = 0.415–1.453, *p*-value = 0.427), showing no differences between both genotypes and tuberculosis resistance. These results suggest that only one copy of the A allele could be enough to confer protection against tuberculosis, in line with a dominant genetic model. Actually, OR were calculated in the dominant and recessive models (Figure 1C), and an association with tuberculosis resistance was found in the dominant model (OR = 0.270; 95% CI = 0.075–0.753; *p*-value = 0.022). Taken together, these data demonstrate an association between the A allele and the AA and AG genotypes with a reduced frequency of individuals suffering from tuberculosis, suggesting a potential relationship between the A allele of the *IFNG* rs1861494 SNP and protection against tuberculosis in Argentina.

In order to get further evidences that support the protective role of the A allele in developing tuberculosis, IFNG levels in plasma and IFNG production by *Mtb*-Ag-stimulated PBMCs from HD carrying the different genotypes of the *IFNG* rs1861494 SNP were determined by ELISA and Flow Cytometry. As shown in Table 3, no differences were found in the IFNG plasmatic levels measured among HD carrying the different genotypes of the *IFNG* rs1861494 SNP. Nevertheless, *Mtb*-Ag-stimulated PBMCs from AA HD displayed the highest IFNG levels in culture supernatants and also presented the greatest percentage of CD4^+^IFNG^+^ lymphocytes, as compared to GG HD subjects. These data are in agreement with the protective role found for the A allele in developing tuberculosis. Furthermore, our findings suggest that AA individuals present the lowest susceptibility to tuberculosis disease probably because they generate a more effective immune response against the bacteria, in comparison with GG carriers.

### 3.3. Lack of Association between the IFNG rs1861494 SNP and the Severity of the Disease

We next analyzed some immunological and clinical parameters in the TB population in order to investigate the potential association between the *IFNG* rs1861494 SNP and the severity of the disease. No differences were found in the plasmatic levels of IFNG, the production of IFNG and the proliferative response of *Mtb*-Ag-stimulated PBMCs in TB carrying the different genotypes variants (Table 3). In addition, no differences between the polymorphism distribution and clinical parameters of the disease severity (hematologic counts, Acid-Fast Bacilli (AFB) in sputum smear or radiological lessons) were detected (Table 4). Therefore, no evidence relating the *IFNG* rs1861494 SNP with a higher tuberculosis severity in the Argentinean population was found.

## 4. Discussion

To date, only two reports in the Argentinean population have analyzed the association between a cytokine SNP and tuberculosis, but no one in the *IFNG* gene, a crucial cytokine for controlling *Mtb* infection [27,32]. Here, by using a case-control study, we reported an association between the AA and AG genotype of the *IFNG* rs1861494 SNP with tuberculosis resistance in Argentina, indicating that only one copy of the A allele is enough to confer protection against the disease in our population. In particular, the *IFNG* rs1861494 SNP was a good candidate to study the genetic association because it has been widely validated (sequenced in 1000 Genome project, Genotyped by HapMap project, and multiple, independent submissions to the ref SNP cluster) [33] and associated with different diseases immunoglobulin A (IgA) nephropathy, vitiligo, inflammatory bowel disease, leprosy, tuberculosis, between others) [28,34,35,36,37].

Other studies have investigated the association between *IFNG* rs1861494 SNP and tuberculosis risk, but inconsistent results were found [37,38,39,40,41]. In contrast to our present findings, the A allele was reported to be related to tuberculosis susceptibility in an Indian population [38]. Also, another study from Taiwan found that AA and AG carriers were risky genotypes regarding susceptibility to tuberculosis [39]. Besides, one report from Croatia and another from China evidenced no association between the SNP and the disease [40,41]. Nevertheless, our results are in agreement with a study performed in Iran, a population ethnically different from ours, further supporting the fact of a higher resistance to tuberculosis disease in individuals carrying the A allele of the *IFNG* rs1861494 SNP [37]. These discrepancies might be related to differences in the selection of the control group, ethnic differences or lack of HW equilibrium in the population analyzed.

Ethnicity and sex could be confounding variables in genetic association analyses. Both populations under study in the present work were comparable in terms of ethnic composition but displayed different sex proportions. Importantly, these ethnic frequencies were quite similar to the ethnic composition of the Argentinean population [42,43]. Remarkably, no differences were found in the genotypic frequencies distribution of the *IFNG* rs1861494 SNP in HD and TB population stratified by ethnicity and sex. Additionally, the genotypes distribution in the HD and TB populations showed no deviation from HW equilibrium, indicating that there is a population substructure. Also, the logistic regression used to evaluate the association between the *IFNG* rs1861494 SNP and tuberculosis disease was adjusted for age, ethnicity and sex. For all the mentioned reasons, it is unlikely that the ethnicity and sex act as confounding variables in these genetic association analyses.

It was evidenced that the rs1861494 SNP might have a functional consequence on *IFNG* expression, as a result of an introduction of a new potential CpG methylation site that altered the binding of transcription factors (Nuclear factor of activated T-cells (NFAT), nuclear factor kappa-light-chain-enhancer of activated B cells (NFκB)) to this region. In fact, it was reported the association of the A allele with higher levels of IFNG [28,29]. We did not find any differences in the plasmatic IFNG among individuals carrying the different genotypes of the *IFNG* rs1861494 SNP, but we did demonstrate that *Mtb*-Ag-stimulated PBMCs from AA HD displayed the highest IFNG levels in culture supernatants and also showed the greatest percentage of CD4^+^IFNG^+^ lymphocytes, in comparison with GG carriers. These data reinforce the idea that AA HD may present the lowest susceptibility to tuberculosis disease by producing the highest levels of IFNG, that may contribute to eliminate the pathogen at a first contact. In contrast to the results obtained in the case-control study, no differences were found between the IFNG production by *Mtb*-Ag-stimulated PBMCs from HD carrying the AG or GG genotype. These data suggest that there would be no differences in the immune response against *Mtb* in HD carrying those genotypes, although a trend to a higher IFNG production was observed in individuals carrying the AG genotype. Studies with a larger sample size, using stimulated pleural fluid mononuclear cells or *Mtb*-specific antigens as stimuli could help to clarify this point.

To our knowledge, this is the first study that evaluates the IFNG production by PBMC stimulated with *Mtb*-Ag from individuals carrying the different genotypes of the *IFNG* rs1861494 SNP. However, no differences in IFNG production by *Mtb*-Ag-stimulated PBMCs were detected in TB carrying the different genotypes variants. Yet, we have previously shown that *Mtb*-infection status is related to a decrease in IFNG production in TB as compared to HD [44,45], probably due to different evasion strategies of the mycobacteria [46]. Especially, it was demonstrated that NFAT plays a critical role in immune containment of tuberculosis disease in vivo and that T cell terminal signaling events, like NFAT and NFκB binding, are altered by *Mtb* antigens, an evasion mechanism that could be involved in T-cell impaired functions during the progression of the disease [47]. These alterations could explain the lack of association between the *IFNG* rs1861494 SNP genotype and IFNG production in TB. Further studies are required to confirm this hypothesis. We also investigated relevant tuberculosis clinical parameters in the different genotypes to analyze whether they could be related to disease severity. However, no differences were found in the hematologic counts, the number of AFB in sputum smear or in the radiological lesions, as previously reported [40].

The possible involvement of another functional polymorphism in strong linkage disequilibrium (LD) with the *IFNG* rs1861494 polymorphism cannot be ruled out. In particular, the rs1861493 SNP (T/C) on the *IFNG* gene has been described to be in LD with rs1861494 SNP, and it is associated with some diseases, like Kawasaki Disease, in which IFNG production plays an important role in its immunopathogenesis [48,49]. Besides, *IFNG* rs1861493 SNP and rs1861494 SNP were associated with susceptibility to pulmonary tuberculosis, but the contribution of each one was not clarified [38]. We cannot exclude the possibility of linkage disequilibrium between the *IFNG* rs1861494 SNP variants and other neighboring polymorphisms without additional fine-mapping analysis of SNPs throughout the region. Nevertheless, the functional mechanism of the *IFNG* rs1861494 SNP, altering transcription factors binding in a regulatory region of the *IFNG* gene, emphasizes the importance of this polymorphism. However, due to the weak effect of a single genetic polymorphism, other genes in the immunity pathway, together with environmental factors, should also be considered.

Overall, this is the first report demonstrating that the *IFNG* rs1861494 SNP is related to tuberculosis resistance in the Argentinean population, with no association with the disease severity. Also, we evidenced that *Mtb*-Ag stimulated PBMCs from HD carrying the AA genotype produce significant higher levels of IFNG in comparison with GG carriers. Therefore, our data suggest that the A allele of the *IFNG* rs1861494 SNP could be considered as a biomarker for tuberculosis resistance in Argentina.

## Figures and Tables

**Table 1:** Demographic characteristics of healthy donors (HD) and tuberculosis (TB) populations.

		HD	TB	*p*-value
*N*		175	201	
**Age (years)**		34.25 ± 1.16	33.03 ± 0.98	0.123 ^a^
**Ethnicity**	Caucasian	65.3%	63.9%	0.896 ^b^
	American Indian	34.7%	36.1%	
**Sex**	Male	36.6%	72.9%	<0.001 ^b^
	Female	63.4%	27.1%	

Categorical variables are expressed in percentages. Age value is expressed as mean ± standard error of the mean (SEM). ^a^ *p*-values were calculated by the Mann-Whitney U test for unpaired samples. ^b^ *p*-values were calculated by Χ^2^ test for categorical variables. *N*: number of individuals.

**Table 2:** Genotypic frequencies distribution of the Interferon gamma (*IFNG*) rs1861494 SNP in HD and TB populations stratified by ethnicity and sex.

rs1861494 Genotypes		HD (*N* = 175)			TB (*N* = 201)		
		GG	GA	AA	GG	GA	AA
**Ethnicity**	Caucasian	9 (7.89%)	62 (54.39%)	43 (37.72%)	20 (15.63%)	59 (46.09%)	49 (38.28%)
	American Indian	6 (9.84%)	30 (49.18%)	25 (40.98%)	17 (23.29%)	34 (46.58%)	22 (30.14%)
	*p*-value	0.783			0.308		
**Sex**	Male	7 (11.11%)	29 (46.03%)	27 (42.86%)	32 (22.07%)	71 (48.97%)	42 (28.96%)
	Female	11 (9.82%)	48 (42.86%)	53 (47.32%)	8 (14.29%)	26 (46.43%)	22 (39.28%)
	*p*-value	0.847			0.268		

The Table shows the number of individuals and frequencies (in parentheses) in HD and TB for each subgroup. *p*-values were calculated by the Χ^2^ test for categorical variables.

**Table 3:** Association between the *IFNG* rs1861494 single nucleotide polymorphism (SNP) genotypic variants and IFNG production in the context of tuberculosis.

	rs1861494 SNP Genotypes			*p*-value
	GG	AG	AA	
**HD (*N* = 21)**				
**IFNG levels in plasma (pg/mL)**	13.59 (±5.78)	9.44 (±1.64)	15.33 (±6.72)	0.797
**IFNG levels in culture supernatants (pg/mL)**	5185 (±1491)	14,195 (±3835)	20,989 (±5218)	0.034
% CD4^+^ T cells IFNG^+^	9.06 (±2.08)	15.47 (±3.66)	20.44 (±2.48)	0.035
**TB (*N* = 21)**				
**IFNG levels in plasma (pg/mL)**	67.27 (±59.21)	46.31 (±20.25)	170.5 (±68.92)	0.144
**IFNG levels in culture supernatants (pg/mL)**	6772 (±2750)	7575 (±2011)	15,066 (±4553)	0.357
% CD4^+^ T cells IFNG^+^	7.91 (±1.38)	12.39 (±4.99)	15.97 (±4.29)	0.809

IFNG production was determined in plasma samples and cell lysate *Mycobacterium tuberculosis* H37Rv strain *(Mtb*-Ag)-stimulated peripheral blood mononuclear cells (PBMC) from HD and TB carrying the different genotypes of the rs1861494 SNP by ELISA and Flow Cytometry. Values are expressed as the Mean ± SEM. *p*-values were calculated by Analysis of variance (ANOVA) and the Kruskal-Wallis post-test for unpaired and non-parametric samples. % CD4^+^ T cells IFNG^+^: percentage of total CD4 positive T cells producing IFNG.

**Table 4:** Association between the *IFNG* rs1861494 SNP genotypic variants and some clinical parameters.

	rs1861494 SNP Genotypes			*p*-value
	GG	AG	AA	
**Hematologic Studies (*N* = 105)**				
**Leucocytes (cells/mL)**	10,754 (±1557)	10,205 (±388.8)	9929 (±702.1)	0.432 ^a^
**Lymphocytes (cells/mL)**	1964 (±349.5)	1491 (±76.45)	1573 (±126.5)	0.762 ^a^
**Monocytes (cells/mL)**	1013 (±138.0)	809.3 (±58.38)	878.6 (±83.13)	0.584 ^a^
**Neutrophils (cells/mL)**	8097 (±1557)	7524 (±586.6)	5674 (±574.4)	0.089 ^a^
**AFB in sputum smear (*N* = 150)**				
**BAAR^−^ or BAAR^+^**	14 (18.0%)	43 (55.1%)	21 (26.9%)	0.415 ^b^
**BAAR^++^ or BAAR^+++^**	17 (23.6%)	32 (44.4%)	23 (32.0%)	
**Radiological Lesions (*N* = 136)**				
**Mild or Moderate**	11 (21.1%)	29 (55.8%)	12 (23.1%)	0.367 ^b^
**Severe**	17 (20.3%)	39 (46.4%)	28 (33.3%)	
**Months of disease progression (*N* = 105)**	2.15 (±0.29)	2.92 (±3.56)	2.29 (±0.35)	0.447 ^a^

Hematologic studies representing the leukocyte, lymphocyte, monocyte and neutrophil counts in peripheral blood are shown. Acid-Fast Bacilli (AFB) in sputum smear represent: BAAR^−^: 0 bacilli count; BAAR^+^: 1–9 bacilli/100 fields; BAAR^++^: 1–9 bacilli/10 fields; BAAR^+++^: 1–9 bacilli/field. Radiological lesions: mild corresponds to patients with a single lobe involved and without visible cavities; moderate relates to patients presenting unilateral involvement of two or more lobes with cavities, if present, reaching a total diameter no greater than 4 cm; severe corresponds to bilateral disease with massive affectation and multiple cavities. Clinical symptoms analyzed in TB previous to hospital admission to establish the time (months) of disease progression were: weight loss, night sweats, symptoms of malaise or weakness, persistent fever, presence of cough, history of shortness of breath, and hemoptysis. Continuous data are expressed as the Mean ±SEM, and categorical data are expressed as number (percentages of genotype). ^a^ *p*-values were calculated by the Kruskal-Wallis (ANOVA) test for unpaired and non-parametric samples. ^b^ *p*-values were calculated by the Χ^2^ test for categorical variables.

## Supplementary Materials

The following are available online at http://www.mdpi.com/2073-4425/9/1/46/s1. Supplementary Figure S1: IFNG rs1861494 SNP genotyping by ARMS-PCR method.
