# Supplementary material for: The IFNG rs1861494 Single Nucleotide Polymorphism Is Associated with Protection against Tuberculosis Disease in Argentina
Source: Genes (Basel). 2018 Jan 22;9(1):46. doi: 10.3390/genes9010046 (PMC5793197; doi:10.3390/genes9010046)
Supplement: Supplementary file 1 [file genes-09-00046-s001.pdf]

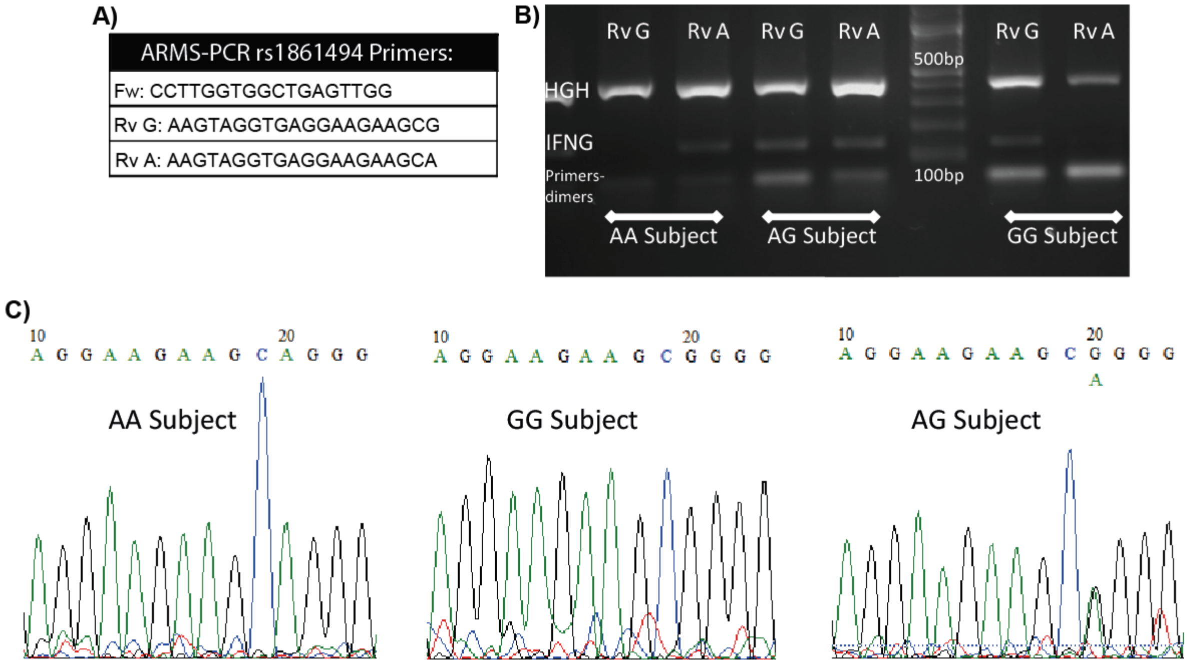

Supplementary Figure S1. ***IFNG*** **rs1861494 SNP genotyping by ARMS-PCR method. (A)** Primer sequences (two Reverse primers and one common Forward primer) designed to specifically amplify a 168 bp amplicon that discriminates both alleles of the *IFNG* rs1861494 SNP. **(B)** Image of an agarose gel displaying the PCR products obtained from three genotypically different individuals for the SNP under study is shown. PCR positive control: Human Growth Hormone (HGH) gene fragment (440 bp). *IFNG* rs1861494 genotypes were assessed from the presence/absence of PCR amplicon corresponding to the specific allele (A or G). **(C)** DNA sequencing of the amplicons obtained from three genotypically different individuals. Primers specificity of the rs1861494 SNP were confirmed by direct sequencing of the amplified *IFNG* gene fragment by Sanger method and a 100% concordance was obtained among the results obtained from ARMS-PCR and DNA sequencing.
